# The change of paradigm in the treatment of HER2-positive breast cancer with the development of new generation antibody-drug conjugates

**DOI:** 10.20517/cdr.2022.52

**Published:** 2023-01-12

**Authors:** Santiago Escrivá-de-Romaní, Cristina Saura

**Affiliations:** Medical Oncology Service, Breast Cancer Unit, Vall d’Hebron Institute of Oncology (VHIO), Barcelona 08035, Spain.

**Keywords:** Breast cancer, HER2-positive, ADCs, New drugs, Mechanisms of resistance

## Abstract

HER2-positive breast cancer is an aggressive disease. As a result of the development of specific HER2-targeted therapies, such as trastuzumab, more than 20 years ago, the prognosis of these patients has improved. Metastatic HER2-positive breast cancer patients are achieving better survival rates upon treatment with anti-HER2 therapies than patients with HER2-negative disease. Double HER2 blockade with trastuzumab and pertuzumab combined with a taxane achieved an unprecedented survival of over 57 months in first-line patients. Trastuzumab emtansine, the first antibody-drug conjugate approved for patients in second-line treatment was a potent cytotoxic agent bound to trastuzumab and is currently a standard therapeutic strategy. Despite the progress in treatment development, most patients develop resistance and eventually relapse. Advances in the design of antibody-drug conjugates have led to the development of new generation drugs with enhanced properties, such as trastuzumab deruxtecan and trastuzumab duocarmazine, which are significantly changing the paradigm in the treatment of HER2-positive metastatic breast cancer.

## INTRODUCTION

### The development of anti-HER2 therapies: from antibodies to antibody-drug conjugates

Human epidermal growth factor receptor 2 protein (HER2)-positive breast cancer is an aggressive disease that accounts for approximately 15%-20% of the total breast cancer cases worldwide^[1]^. Most of these cases are diagnosed at the early stage. The HER2 pathway drives the growth and expansion of tumor cells. Research into the development of anti-breast cancer therapies has been focused on blocking the HER2 receptor with different strategies. 

### Trastuzumab

Trastuzumab is an anti-HER2 humanized monoclonal antibody directed against the extracellular portion of HER2. Trastuzumb was the first therapy approved by the FDA in 1998 for metastatic breast cancer patients with tumors with HER2 overexpression and it was approved in 2006 for use in the adjuvant setting after demonstrating a significant benefit in progression-free survival (PFS) and overall survival (OS) in both early and advanced disease^[2,3]^. The mechanisms of action of trastuzumb are the inhibition of HER2 shedding and inhibition of the PI3K-AKT pathway, resulting in an attenuation of cell signaling and promotion of antibody-dependent cell-mediated cytotoxicity (ADCC). Trastuzumab has significantly changed the landscape for HER2-positive patients^[4]^. However, some tumors acquire resistance to trastuzumab through mechanisms including increased cell signaling, PTEN loss, PIK3CA mutations, increased AKT activity, alternative cell signaling mediated by EGFR pathways, TGF-α overexpression, and expression of extracellular domain-truncated HER2 (p95 HER2)^[5]^.

### Pertuzumab

To overcome trastuzumab resistance and improve treatment efficacy, a strategy was developed of combining trastuzumab with pertuzumab, another antibody that binds a different epitope of HER2, preventing the formation of HER2-HER3 heterodimers, the most active forms in signaling^[6]^. The combination of this double anti-HER2 blockade with a taxane was approved by the FDA in 2012 for first-line treatment for HER2-positive advanced disease on the basis of results of the CLEOPATRA trial, which demonstrated an improvement in PFS and OS with this strategy^[7]^.

### Trastuzumab-emtansine (T-DM1): the first anti-HER2 ADC

**Figure 1:**
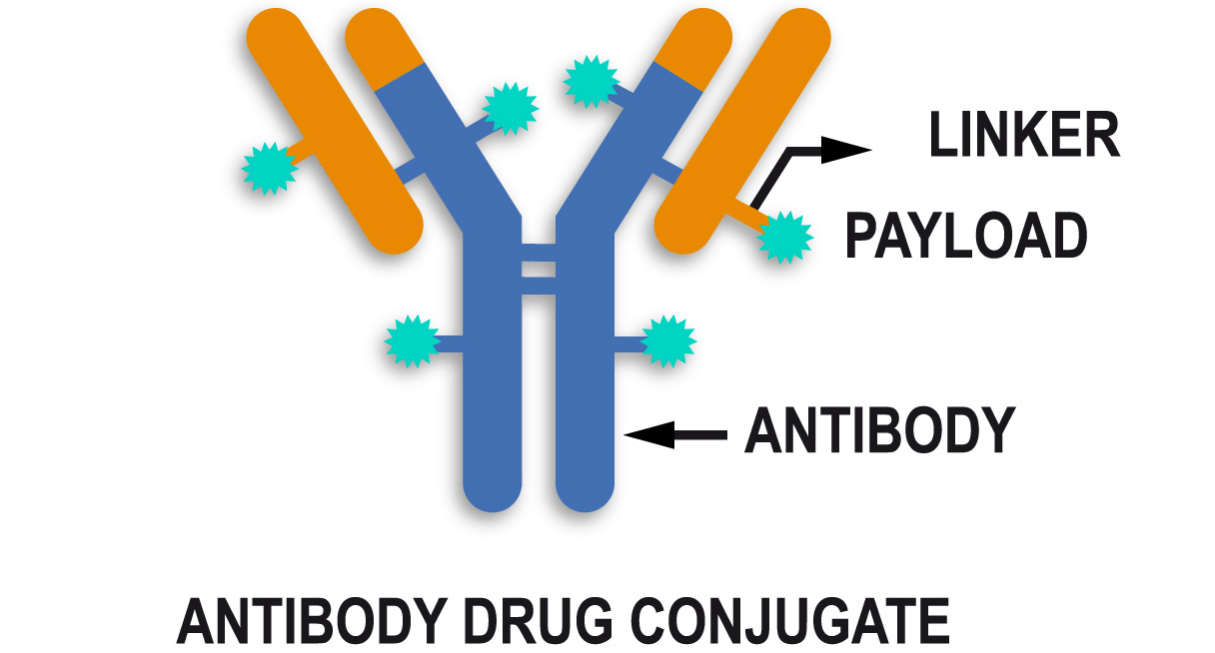
General structure of an antibody drug conjugate. The antibody drug conjugate contains three key components: antibody, linker, and payload.

Through continued research, another strategy was developed using a novel drug design technology for antibody-drug conjugates (ADCs), in which a potent cytotoxic agent is conjugated to an antibody with a linker to selectively deliver the payload to cells expressing a specific antigen, theoretically sparing normal cells from toxicity [Figure 1]. HER2-positive disease was an attractive target for ADC development. T-DM1, the first ADC, was designed to act against the HER2 receptor [Table 1]. The clinical benefit of T-DM1 in terms of both efficacy and toxicity was demonstrated in the EMILIA trial, which compared T-DM1 with the combination of capecitabine and the tyrosine kinase inhibitor (TKI) lapatinib. The results showed a significant improvement in PFS and OS with T-DM1, leading to the approval of T-DM1 by the FDA in 2013^[8,9]^. Later attempts to position T-DM1 in advanced first-line treatment or even in the neoadjuvant setting were not so successful, as there was no significant benefit in first-line treatment with T-DM1 compared with trastuzumab and a taxane or in the neoadjuvant early setting comparing TM-1 combined with pertuzumab and TCHP (docetaxel, carboplatin, trastuzumab, pertuzumab)^[10,11]^. However, in the KATHERINE trial, T-DM1 significantly decreased the risk of relapse in patients who had remaining residual disease after anti-HER2 neoadjuvant treatment^[12]^.

**Table 1:** T-DM1 main phase 3 trials

**ADC**	**Characteristics**	**Trial/Population**	**Design**	**Results**
Ado-trastuzumab emtansine T-DM1	Payload: DM1 DAR: 3,5 Linker: non-cleavable thioether Bystander effect: no	EMILIA^[8,9]^: HER2+ MBC previously treated taxane + trastuzumab	T-DM1 *vs.* Capecitabine + Lapatinib (C + L)	T-DM1 *vs.* C + L PFS: 9.6 m *vs.* 6.4 m. HR 0.68 (0.55-0.86) OS: 30.9 m *vs.* 25.1 m. HR 0.65 (0.55-0.77)
		TH3RESA: HER2+ MBC previously treated taxane, trastuzumab, lapatinib	T-DM1 *vs. *TPC	T-DM1 *vs.* TPC PFS: 6.2 m *vs.* 3.3 m. HR 0.53 (0.53-0.66) OS: 22.7 m *vs.* 15.8 m. HR 0.68 (0.54-0.85)
		MARIANNE^[10]^: HER2+ previously untreated MBC	Trastuzumab + taxane *vs.* T-DM1 *vs.* T-DM1 + pertuzumab	T-DM1 *vs.* Trastuzumab + taxane PFS: 14.1 m *vs.* 13.7 m. HR 0.91 (0.73-1.13) T-DM1 + pertuzumab *vs.* Trastuzumab + taxane PFS: 15.2 m *vs.* 13.7 m. HR 0.87 (0.69-1-08)
		KATHERINE^[12]^: HER2+ early disease. Residual invasive disease after neoadjuvant treatment with a taxane + trastuzumab	T-DM1 *vs.* Trastuzumab	TDM1 *vs.* Trastuzumab IDFS: 87.8% *vs.* 77.8%. HR 0.50 (0.39-0.64) OS: 94.35 *vs.* 92.5%. HR 0.70 (0.47-1.05)

ADC: Antibody-drug conjugate; DAR: drug-to-antibody ratio; MBC: metastatic breast cancer; m: month; HR: hazard ratio; PFS: progression-free survival; OS: overall survival; TPC: treatment of physician’s choice; IDFS: invasive disease free survival.

**Figure 2:**
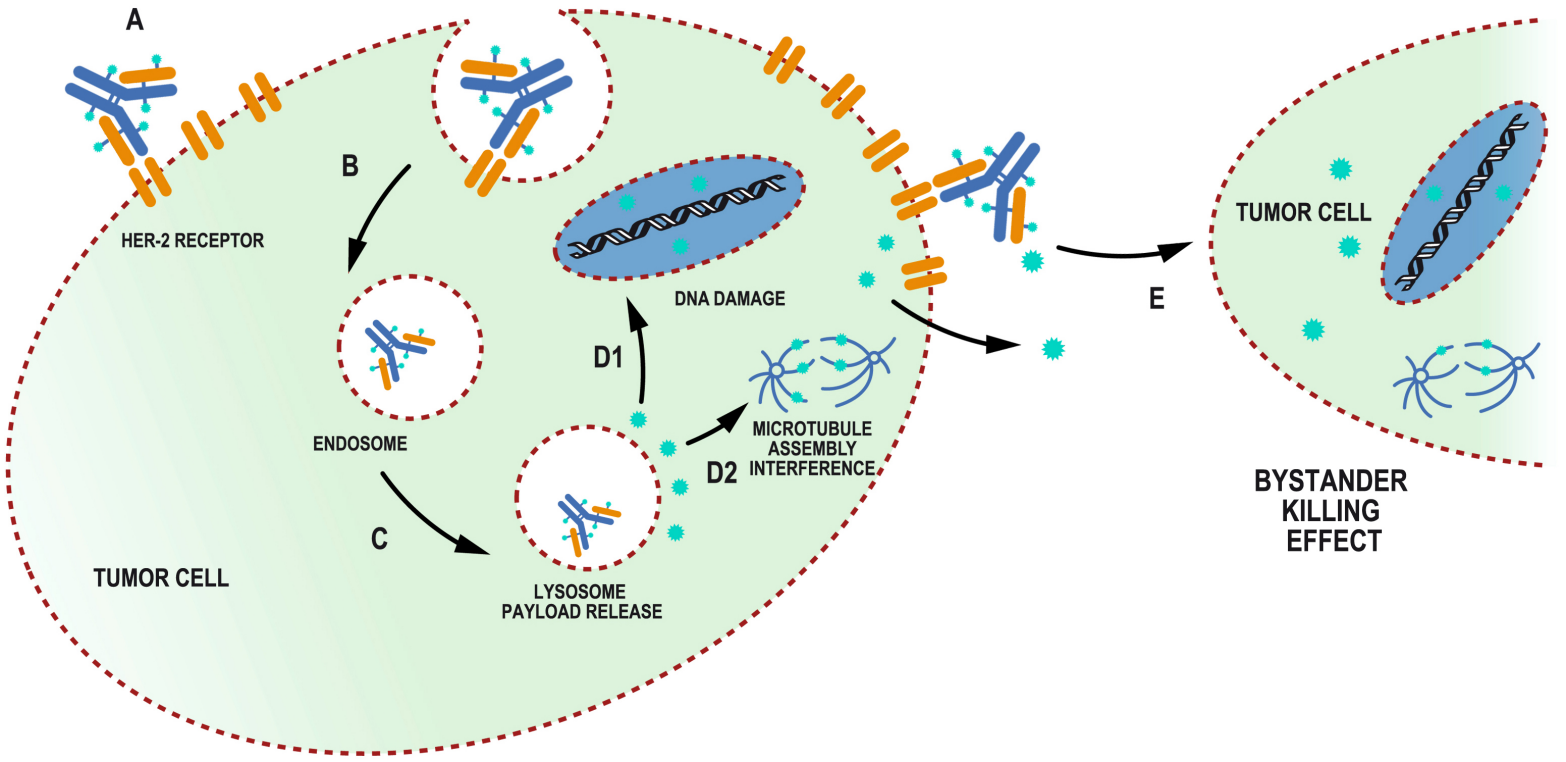
General mechanism of action of an antibody drug conjugate (ADC). A: The antibody binds to the HER2 receptor (RT) in the tumor cell and initiates the internalization process. B: The ADC-RT complex forms an endosome. C: Lysosomal degradation of the endosome releases the payload to the cytoplasm. D: The payload produces various cytotoxic effects, such as the following: D1, DNA damage, as with the topoisomerase I inhibitor in trastuzumab deruxtecan; D2, microtubule assembly interference, as with DM1 in trastuzumab emtansine; and E, bystander killing effect in neighbor tumor cells not expressing the antigen. After payload liberation in the tumor microenvironment by proteases, the payload, with cell membrane permeability, exhibits cytotoxic effects in neighboring tumor cells.

A better understanding of the mechanisms of action of T-DM1 will help elucidate how primary and acquired resistance develop *in vivo*, and thus these mechanisms have been a subject of intense investigation^[13]^. The antitumor effects of T-DM1 reflect the activities of its components. Trastuzumab not only enables binding to tumor cells, but also inhibits HER2 signaling, HER2 extracellular domain shedding and ADCC. DM1 is a potent derivative of the maytansinoid toxin with a cytotoxic effect that is mediated through the inhibition of tubulin polymerization, which leads to the death of proliferating cells. Another key component is the non-cleavable thioether linker that conjugates trastuzumab and its payload, which allows the release of DM1 through liposomal degradation after the receptor-ADC complex has been internalized in the cell [Figure 2]. The drug-to-antibody ratio (DAR) for T-DM1 is 3.5:1 which defines the number of cytotoxic payloads held by each antibody. This is an important characteristic of an ADC that may be related to its potency^[14]^. Once the active payload lysine-MCC-DM1 complexes are released from the lysosome, cytotoxic effects are induced in the tumor cell but not in neighboring cells because of the membrane impermeability of the complexes^[15]^. Hence, there is no bystander effect from the killing of nearby tumor cells that do not present the antigen. Notably, loss or reduction of HER2 expression disables the internalization of ADC and thus represents the main mechanism of resistance^[16,17]^. Intratumor heterogeneity of HER2 expression leads to reduced access of T-DMI to non-HER2-expressing cells and might influence primary resistance to T-DM1, as has been suggested in clinical trials^[18,19]^. Alterations in the internalization of the receptor-T-DM1, formation of endosomes and the lysosome pathway required for release of the DM1 payload have also been described in T-DM1 resistant cell lines in preclinical studies^[13]^. The upregulation of drug efflux transporters of which T-DM1 and other maytansinoids are substrates may also lead to resistance^[20]^. Other *in vitro* studies suggest that resistance to DM1-mediated cytotoxicity may result from the reduction of cyclins needed to promote cell progression to the mitotic phase, causing attenuation of mitotic catastrophe and apoptosis^[21]^.

One of the strategies proposed to overcome or delay resistance to T-DM1 is the combination of T-DM1 with other drugs. The combination of T-DM1 with the anti-PD-L1 antibody atezolizumab was evaluated in the phase II trial KATE2 and showed improved PFS for the PD-L1-positive population but negative results for the intention-to-treat population^[22]^. The subsequent clinical trial, KATE3, is recruiting HER2+/PD-L1-positive patients with advanced breast cancer and will explore the efficacy of the combination. Furthermore, the Astefania study is examining the efficacy of the combination of T-DM1 and atezolizumab in patients with high-risk (N+) residual disease after neo-adjuvant chemo/trastuzumab/pertuzumab^[23]^.

The combination of T-DM1 with anti-HER2 TKIs is another potential treatment strategy to improve efficacy and takes advantage of different anti-HER2 mechanisms of action as well as increasing activity in the brain since TKIs are able to cross the brain-blood-barrier. The HER2CLIMB 02 trial phase III is exploring the combination of T-DM1 with the reversible anti-HER2 TKI tucatinib^[24]^.

## NEW GENERATION OF ADCs

As another potential strategy to overcome resistance, a new generation of anti-HER2 ADCs, such as trastuzumab deruxtecan (T-DXd) and trastuzumab duocarmazine (SYD985), with an enhanced design that confers improved efficacy, is a current focus of research. There are new generation ADCs that are similar to T-DM1, as they use antibodies with the same sequence as trastuzumab, thus targeting the same antigen [Tables 2 and 3]. This is the case for trastuzumab deruxtecan and trastuzumab duocarmazine. Others, like ARX-788, use a different anti-HER2 antibody to bind to tumor cells. These ADCs also show differences in the linker technology design that permits them to be cleavable under specific conditions and do not require lysosomal degradation, as in T-DM1. The cytotoxic payloads have different mechanisms of action and properties, such as membrane permeability, which allows for killing of neighbor cells not presenting the antigen, enhancing the efficacy of these agents [Figure 2]. These properties also result in activity in tumors expressing lower levels of HER2 (defined as HER2-low tumors) and enable the treatment of HER2-negative patients that might also obtain clinical activity from these ADCs^[25]^. 

**Table 2:** New generation anti-HER2 ADCs main trials in HER2-positive population with available results

**ADC**	**Characteristics **	**Main trials in MBC**	**Design**	**Available results**
Trastuzumab deruxtecan T-DXd	Payload: exatecan derivative DAR: 7,7 Linker: cleavable Bystander effect: yes	DESTINY-Breast 01^[31,32]^ Phase 2: HER2+ MBC previously treated with T-DM1 NCT03248492	Single arm T-DXd	ORR: 61.4% PFS: 19.4 m (14.1-NE) OS: 24.6 m (23.1-NE)
		DESTINY-Breast 03^[33]^ Phase 3: HER2+ MBC previously treated taxane + trastuzumab NCT03529110	T-DXd *vs.* T-DM1	T-DXd *vs.* T-DM1 ORR: 79.7% *vs.* 34.2% PFS: NR *vs.* 6.8 m. HR 0.28 (0.22-0.37) OS: NR *vs.* NR. HR 0.56 (0.36-0.86) 12 m OS: 94.1% *vs.* 85.9%.
Trastuzumab duocarmazine SYD985	Payload: vc-seco-DUBA DAR: 2.8 Linker: cleavable valine-citruline Bystander effect: yes	TULIP^[41]^ Phase 3: HER2+ MBC previously treated with 2 lines or T-DM1 NCT03262935	SYD985 *vs.* TPC: Capecitabine + trastuzumab/lapatinib or Vinorelbine + trastuzumab or eribuline + trastuzumab	SYD985 *vs.* TPC PFS: 7 m *vs.* 4.9 m. HR 0.64 (0.49-0.84) OS: 20.4 m *vs.* 16.3 m. HR 0.83 (0.62-1.09)
ARX788	Payload: dolastatin monomethyl auristatin F Linker: non-cleavable AS269 DAR: 1.9 Bystander effect: no	ACE-Pan Tumor 01^[43] ^ Phase 1: advanced solid tumors with HER2 expression NCT03255070	ARX788	Breast cohort: PFS 17 m
RC48-ADC	Payload: monomethyl auristatin E DAR: 4 Linker: cleavable valine-citruline Bystander effect: no	Phase 1^[45]^: advanced breast cancer with HER2+ or HER2 low expression NCT03052634	RC48-ADC	Pooled results in HER2+ MBC: PFS: 4 to 6.3 m
ZW49	Payload: N-acyl sulfonamide auristatin DAR: 2 Linker: cleavable Bystander effect: NA	Phase 11^[51]^: HER2 expressing cancers NCT03821233	ZW49	Results in HER2+ MBC: ORR: 13% PFS: NA

ADC: Antibody-drug conjugate; DAR: drug-to-antibody ratio; MBC: metastatic breast cancer; NA: non available; ORR: objective response rate; m: month; HR: hazard ratio; PFS: progression-free survival; OS: overall survival; TPC: treatment of physician’s choice.

**Table 3:** New generation anti-HER2 ADCs main trials in HER2-positive populationin progress

**ADC**	**Characteristics**	**Main trials in MBC**	**Design**	**Available results**
Trastuzumab deruxtecan T-DXd	Payload: exatecan derivative DAR: 7,7 Linker: cleavable Bystander effect: yes	DESTINY-Breast 02 Phase 3: HER2+ MBC previously treated with T-DM1 NCT03523585	T-DXd *vs.* TPC (Capecitabine + trastuzumab or lapatinib)	Results pending
		DESTINY-Breast 09 Phase 3: HER2+ previously untreated MBC NCT04784715	T-DXd + placebo *vs.* T-DXd + pertuzumab *vs.* Taxane + trastuzumab + pertuzumab	Recruiting
		DESTINY-Breast 05 Phase 3: HER2+ early disease. Residual invasive disease after neoadjuvant treatment with a taxane + trastuzumab NCT04622319	T-DXd *vs.* T-DM1	Recruiting
		DESTINY-Breast 11 Phase 3: HER2+ early disease neoadjuvant treatment NCT05113251	T-DXd *vs.* T-DXd - Taxane + trastuzumab + pertuzumab (THP) *vs.* dose dense AC - THP	Recruiting
Trastuzumab duocarmazine SYD985	Payload: vc-seco-DUBA DAR: 2.8 Linker: cleavable valine-citruline Bystander effect: yes	BYON5667.002 Phase I/II NCT04983238	SYD985+BYON5667(eye-drops)/placebo to reduce ocular toxicity	Results pending
ARX788	Payload: dolastatin monomethyl auristatin F Linker: non-cleavable AS269 DAR: 1.9 Bystander effect: no	ACE-Breast-03 Phase 2: HER2+ MBC resistant/refractory to T-DM1, and/or T-DXd, and/or Tucatinib NCT04829604	ARX788	Recruiting
RC48-ADC	Payload: monomethyl auristatin E DAR: 4 Linker: cleavable valine-citruline Bystander effect: no	Phase 2/3: HER2 + MBC with/without liver metastases NCT03500380	RC48-ADC *vs.* capecitabine + lapatinib	Recruiting
ZW49	Payload: N-acyl sulfonamide auristatin DAR: 2 Linker: cleavable Bystander effect: NA	Phase 1: HER2 expressing MBC NCT03821233	ZW49	Recruiting
MEDI4276	Payload: tubulysin-based microtubule inhibitor Linker: cleavable DAR: 4 Bystander effect: yes	Phase 1/2^[41]^: HER2 expressing breast or gastric/stomach cancers NCT02576548	MEDI4276	Completed

ADC: Antibody-drug conjugate; DAR: drug-to-antibody ratio; MBC: metastatic breast cancer; TPC: treatment of physician’s choice; NA: non available.

All these potential improvements are already being translated into a significant clinical benefit in HER2- positive metastatic breast cancer patients, as some of these new ADCs such as T-DXd and trastuzumab duocarmazine are already in an advanced phase of clinical development.

These promising therapies have raised interest in the scientific community, and several recent reviews describing the most updated data in this field have been published^[26-28]^.

### Trastuzumab-Deruxtecan

T-DXd is composed of trastuzumab linked by an enzymatically cleavable peptide-linker to DXd, which is an exatecan derivative, a potent topoisomerase I inhibitor that induces double-strand DNA breaks and apoptosis. The DAR of T-DXd is 7.7:1, which is higher than that of T-DM1 (3.5:1), allowing more delivery of payload by each ADC in target tumor cells. Preclinical results have shown promising antitumor effects in T-DM1-resistant cells and low HER2-expressing cells^[29]^. From these results, the initial clinical development plan targeted HER2-positive patients who had become resistant to T-DM1 or whose tumors were expressing low HER2 [defined as HER2 1+ and 2+ by immunochemistry with in situ hybridization (ISH) negative]. The phase I trial included 115 HER2-positive patients treated with the doses recommended for expansion that had received previous therapy (a median of seven lines); the overall response rate (ORR) was 60% in this heavily pretreated population. The toxicity profile showed more frequently gastrointestinal and hematological adverse events and 20 patients developed interstitial lung disease (ILD), including two treatment-related deaths from pneumonitis^[30]^. The phase 2 trial DESTINY-breast01 confirmed the clinical activity of T-DXd in HER2-positive patients resistant to T-DM1 and confirmed 5.4 mg/kg as the recommended dose for further development in phase III trials. A total of 184 patients received T-DXd at the recommended dose with a median of six previous lines of therapy in the metastatic setting. The primary endpoint was objective response following independent central review with other efficacy and safety secondary endpoints^[31]^. Updated results with a 20.5 median follow-up confirmed a 61.4% ORR and 19.4 months PFS with a median duration of response of 20.8 months. The most common G3 or higher adverse events were neutrophil count decrease, anemia, and nausea. Approximately 15.2% of cases had ILD, and 2.7% (5 patients) died^[32]^. As a result of the clinical activity demonstrated in this phase 2 trial for patients who had developed resistance to previous therapies including T-DM1, T-DXd was approved by the FDA and EMA for use in HER2-positive metastatic patients that had received at least two previous lines of treatment. The most relevant data on the clinical activity of T-DXd reported thus far is the head-to-head comparison with T-DM1 in the phase III DESTINY-breast03 trial. Patients with HER2-positive advanced breast cancer that had been previously treated with a taxane and trastuzuzmab were randomized at 1:1 to receive T-DXd or T-DM1. The primary endpoint was PFS and OS was a key secondary endpoint. The median follow-up for T-DXd was 16.2 months; the median PFS was 6.8 months for the T-DM1 groups, while the median PFS had not been reached for the T-DXd group, with a highly significant difference (HR of 0.28, 0.22-0.37, *P* = 7.8 × 10^-22^). The median OS had not been reached for both treatment arms, with a 12-month OS rate of 94.1% for the T-DXd group and 85.9% for the T-DM1 group, with no significant difference in this first analysis. The difference in ORR between the groups, with 79.7% for the T-DXd group compared with 34.2% for the T-DM1 group, was remarkable. The most common toxicity was hematological and gastrointestinal toxicity, with nausea as the most frequent event. Among the 524 randomized patients, 27 patients (10.5%) had ILD and 2 cases had G3 events with no cases of fatal pneumonitis for T-DXd compared with 5 cases of G1-2 for T-DM1^[33]^. These data currently position T-DXd as a standard second-line treatment in the metastatic setting, moving T-DM1 to later lines^[34]^. Another ongoing clinical trial is exploring the role of T-DXd in first-line treatment; this trial is comparing T-DXd combined with pertuzumab/placebo to the standard treatment of a taxane plus double blockade with trastuzumab and pertuzumab^[35]^. This ADC is likely to make its way to the early setting, because ongoing clinical trials are comparing it to adjuvant T-DM1 in post-neoadjuvant residual disease and another trial is exploring it as initial neoadjuvant treatment alone or in sequence with paclitaxel, trastuzumab and pertuzumab (THP) compared with the sequential standard scheme of anthracycline-cyclophosphamide followed by THP^[36,37]^. Special attention and monitoring of potential T-DX-related ILD is being applied in these early disease trials, with clinical awareness and periodical imaging testing to rule out this potential severe toxicity.

### Trastuzumab duocarmazine 

Trastuzumab duocarmazine (SYD985) is another second-generation ADC that is composed of an antibody backbone with the same amino acidic sequence as trastuzumab with a cleavable linker to the vc-seco-DUBA payload, a potent duocarmacyn analog with a DAR of 2.8:1. The cytotoxic payload is an alkylant agent that binds to the minor groove of DNA and exhibits activity in both dividing and non-dividing cells. The linker is cleaved by proteases of the lysosome after endocytosis and by proteases such as cathepsin B that are found extracellularly; thus, there is a bystander killer effect^[38]^. Preclinical studies that compared SYD985 with T-DM1 demonstrated encouraging activity of SYD985 in HER2-positive and HER2 low expression models, prompting exploration of SYD985 in a phase I trial in breast cancer and other HER2-expressing histologies^[39]^. In breast cancer dose-expansion cohorts, 33% of patients with HER2-positive breast cancer achieved objective partial responses; additionally, 28% of patients with HER2-low, hormone receptor-positive breast cancer and 40% with HER2-low, hormone receptor-negative breast cancer also showed objective partial response. The most common treatment-related adverse events were fatigue, conjunctivitis, and dry eye; most patients had at least one ocular adverse event^[40]^. The drug went directly into the TULIP trial, a phase III trial in a HER2-positive metastatic patient population that compared 2:1 SYD985 with the treatment of physician’s choice (TPC) based on predefined standard options of a combination of chemotherapy and trastuzumab or lapatinib. Patients with two or more previous lines in the metastatic setting or with previous T-DM1 were included; patients had a median of four prior therapies. The primary endpoint of the study was centrally reviewed median PFS, which was 7.0 months for the SYD985 group and 4.9 months for the TPC group, with a statistically significant HR of 0.64 and no benefit in terms of OS in the first analysis. No significant differences were observed in ORR or health-related quality of life (HRQoL). The most frequently reported adverse events for SYD985 were conjunctivitis (38.2%), keratitis (38.2%) and fatigue (33.3%). Approximately 7.6% of patients treated with SYD985 showed ILD/pneumonitis, including two fatal events^[41]^. Ocular toxicity from this ADC may potentially be permanent, so it is crucial to treat it and find strategies to mitigate it.

### ARX788

In addition to the two anti-HER2 ADCs described above, other ADCs are in the earlier phase of development, such as ARX788. This new generation ADC has a site-specific anti-HER2 antibody with an amino acid sequence different from that of trastuzumab; it uses a nonnatural amino acid-enabled conjugation technology and a non-cleavable Amberstatin (AS269) drug-linker, a highly potent tubulin inhibitor with a DAR of 1.9:1. Preclinical data demonstrated activity in HER2-high and HER2-low expression cell lines and xenograft and patient-derived xenograft models of breast and gastric cancer, motivating clinical development in these patient populations^[42]^. The phase I trial ACE-Breast-01 examined the use of ARX788 in HER2-positive metastatic breast cancer patients whose disease was resistant or refractory to HER2-targeted agents. The results showed a disease control of 100% and a median PFS of 17 months in the 29 patients treated at therapeutic doses who had been heavily pretreated in the advanced setting. However, there was some uncertainty about the anti-HER2 therapies to which this population would have been previously exposed. The safety profile was also favorable, with most adverse events being G1-G2; no dose-limiting toxicities were found and no drug-related deaths were reported. Since the cytotoxic payload of ARX788 is not a substrate of common efflux transporters, this is probably not a mechanism of resistance^[43]^. The phase 2 trial ACE-Breast-03 is ongoing and includes patients whose disease is resistant or refractory to T-DM1 and/or T-DXd and/or tucatinib-containing regimens^[44]^.

### RC48-ADC

RC48-ADC is a HER2-targeting ADC with a cleavable linker and a potent microtubule inhibitor payload MMAE with bystander killing effect in tumor cells. The DAR for this ADC is 4:1. Pooled results from two phase I studies in HER2-positive and HER2-low patients showed that among 70 HER2-positive patients, the ORR ranged from 22.2%-42.9% and the median PFS was 4-6.3 months for the different dosing levels. The most frequent G3 and above adverse events were decreased neutrophil count, increased GGT and fatigue^[45]^. This ADC is currently in various clinical trials including a phase II/III for HER2-positive patients that is comparing RC48-ADC with capecitabine and lapatinib^[46]^. As the field is moving forward, there are new generation TKIs that are improving the results obtained with lapatinib, hence capecitabine and lapatinib might no longer be considered an optimal control arm in future clinical trials^[47,48]^.

### ZW49 and MEDI4276

Other new generation ADCs include zanidatamab zovodotin (ZW49), which contains a biparatopic antibody to optimize binding to target tumor cells. This ADC consists of the antibody ZW25 (zanidatamab) that binds to the same epitopes of the HER2 receptor as trastuzumab and pertuzumab and a cleavable linker to an aurastatine payload, with a DAR of 2:1. Preclinical data demonstrated antitumor activity in low and high HER2-expressing breast cancer cell lines and PDX models^[49]^. A phase I trial is currently ongoing in patients with locally advanced or metastatic HER2-expressing cancers; additional cohorts are being recruited^[50]^. Preliminary results have suggested promising efficacy in various types of HER2-positive tumors. In eight breast cancer patients with a median of six prior therapies treated at the cohort expansion recommended dose, the ORR was 13%. Toxicity analysis revealed two cases of G2 keratitis lasting more than 14 days; approximately 43% of patients exhibited keratitis, but all events decreased to G1 or eventually resolved. There were no ILD events or deaths related to treatment^[51]^.

Another new generation ADC is MEDI4276, a biparatopic tetravalent antibody targeting two epitopes of the HER2 ecto-domain that has site-specific conjugation to a tubulysin-based microtubule inhibitor payload. Results from a phase I trial in advanced breast and gastric cancer demonstrated clinical activity, but also alteration of liver function tests and gastrointestinal toxicity^[52]^.

ZW49 and MEDI4276 are representative examples of ADCs currently in clinical development for HER2-positive and HER2-low expressing populations, but there are many other ADCs. In the following years, new ADCs targeting HER2 will be developed. While ADCs are demonstrating unprecedented response rates and improvements in PFS that will likely translate in a clinically significant increase in OS, there is an important need to discover the mechanisms underlying the resistance that eventually develop as patients progress. A better understanding of the mechanisms underlying primary and acquired resistance will help inform the development of treatment strategies for patients. Knowledge of the presence of cross-resistance to payloads in different ADCs would help predict the absence of clinical benefit in a patient, thus avoiding unnecessary toxicity.

### Combining ADCs

The combination of ADCs with other agents is a potential strategy to overcome or delay resistance and is being explored in clinical trials. The combination of ADCs with targeted agents is the most widely used approach, especially with TKIs and immune checkpoint inhibitors. Theoretically, these agents could enhance antitumor activity by targeting the intracellular domain of the HER2 receptor and trespassing the blood-brain-barrier (in the case of TKIs) or triggering innate and adaptive immunity (in the case of anti-PDL1 agents). Determining whether this combination strategy leads to improvement in efficacy without significantly increasing toxicity is critical.

### Activity beyond HER2-positive: How low can we go

Research has demonstrated that a higher expression of HER2 corresponds with a greater clinical benefit. HER2 positivity has generally been defined by immunohistochemistry (IHC) and in situ hybridazation (ISH) and used to predict the clinical benefit of anti-HER2 therapies. Notably, the new generation ADCs are also demonstrating significant activity *in vitro* and clinical benefit in patients considered HER2-negative, redefining a so-called HER2-low population that would include tumors with IHC HER2 1+ and HER2 2+ with ISH negative. The threshold of HER2 expression from which a patient might benefit from these agents has not yet been clearly determined. Recent results from the phase II DAISY trial demonstrated activity of T-DXd in heavily pretreated patients with different levels of HER2 expression. Notably, clinical activity was observed in the cohort that included patients with HER2-null tumors (IHC0+)^[53]^. These results suggest that the levels of HER2 expression needed for ADC to exert clinical activity might vary depending on the properties of the different linkers and payloads. The DAISY trial was designed to address the mechanisms of action and resistance to T-DXd. The results indicated that HER2 levels were significantly associated with efficacy, with a median PFS of 11 months for HER2-positive patients compared with 4 months for HER2-null patients. A high percentage and spatial distribution of HER2 IHC0+ cells was associated with a non-response to T-DXd. Approximately 65% of patients progressing to T-DXd had a decrease in HER2 expression compared with baseline levels^[54]^. There was a difference in the transcriptomic response to T-DXd depending on HER2 levels. No recurrent baseline driver mutations were identified as predictors of primary resistance, but 6% of patients presented an ERBB2 hemizygous deletion that might be associated with upfront resistance. SLX4 gene mutations were found in 20% of biopsies tested at progression, suggesting that SLX4 may be involved in a potential mechanism of acquired resistance^[55]^. 

Results from the phase 3 randomized study DESTINY-breast04 were reported in ASCO 2022 for the HER2-low population that accounts approximately for at least 50% of all metastatic breast cancer cases^[25]^. Patients with HR-positive tumors with one or two previous lines of therapy were included and randomized at 2:1 to receive T-DXd or TPC. There was a significant difference in PFS (the primary endpoint) of 10.1 months *vs.* 5.4 months, in favor of T-DXd, with a HR of 0.51. Furthermore, there was also a significant advantage in OS (23.9 months *vs.* 17.5 months) with a HR of 0.64. There was a similar benefit for the smaller exploratory subgroup of HR-negative patients included in the trial. These results led to FDA approval of T-DXd as the first ADC for treatment for a HER2-low population.

### Brain metastases

As the survival of HER2-positive advanced cancer patients increases, the risk of the development of brain metastasis also increases. The activity of ADCs for patients with locally treated brain metastasis has been demonstrated in clinical trials for anti-HER2 ADCs, but there is not a wide representation of this population because of the restrictive inclusion criteria. Exploring how these agents perform in patients with non-treated or active brain disease is critical to design the best therapeutic sequence. Data from the phase IIIb single-arm KAMILLA clinical trial demonstrated the activity of T-DM1 in patients with brain metastases even in the absence of previous local treatment, challenging the hypothesis that larger molecules such as ADCs might not be capable of crossing a non-disrupted blood-brain barrier^[56]^. Preliminary data from DEBBRAH and TUXEDO trials with T-DXd were designed to address this question and demonstrated responses in patients with active brain metastases, both untreated patients and those progressing after previous local treatment^[57,58]^. The ongoing DESTINY-Breast12 trial is enrolling up to 250 patients with either active or stable brain metastases and should shed further light on the role of T-DXd in patients with CNS disease^[59]^.

### Mitigating toxicity

While current data on the potential effects of ADCs are encouraging, there is still room for improvement in terms of efficacy and toxicity. The toxicity profiles are different among ADCs depending on the payload used and the properties of the linkers that are cleavable under specific conditions. A better understanding of the toxicity profile of these agents is required to develop strategies to mitigate the most frequent adverse events along with adverse effects that are rare but might be severe. Identifying the mechanisms and risk factors that favor the occurrence of ILD is important to prevent ILD. A very strong research effort is ongoing, but so far, the most efficacious approach is an early diagnosis through radiological imaging and the identification of respiratory symptoms to enable treatment and initiate ILD management following available guidelines. There is also a need to understand the underlying causes of the eye toxicity that can be found with these agents. In the case of SYD985, activation of the payload in the conjunctive tissue initially produces xerophthalmia, which may evolve into different ocular alterations including keratitis. One possible strategy for avoiding these ocular side effects is already under investigation in a clinical trial that is investigating SYD985 and eye drops specifically developed to inactivate the payload in the eye^[60]^. 

## CONCLUSIONS

The new ADCs are rapidly changing the paradigm of treatment of HER2-positive advanced breast cancer patients and expanding the population that can benefit from them even in patients previously considered HER2-negative that have been defined as HER2-low.The results demonstrating the level of PFS achieved even in heavily pretreated populations indicate that these agents will improve survival. The proportion of HER2-positive metastatic patients that remain in a long-term response that could be considered potentially cured increases as new therapies are being developed, especially with the availability of these new generation ADCs. However, because a considerable number of patients will still eventually show disease progression, there is a need to continue developing more effective therapeutic options. A better understanding of the mechanisms of resistance to these agents is required to develop new strategies to overcome resistance as well as to define the best therapeutic sequence for each patient. Toxicity is still an issue and there is a need for better comprehension of the mechanisms and factors contributing to toxicity to develop mitigating strategies, especially with the implementation of these ADCs in the early stage disease as neo/adjuvant therapies.

## DECLARATIONS

### Copyright

© The Author(s) 2023.
